# Prevalence and molecular characterization of colistin resistance in *Pseudomonas aeruginosa* isolates: insights from a study in Ardabil hospitals

**DOI:** 10.1186/s12866-024-03309-1

**Published:** 2024-05-03

**Authors:** Saghar Jafari-Ramedani, Maryam Nazari, Mohsen Arzanlou, Hadi Peeri-Dogaheh, Amirhossein Sahebkar, Farzad Khademi

**Affiliations:** 1 Department of Microbiology, School of Medicine, Ardabil University of Medical Sciences, Ardabil, Iran; 2 Biotechnology Research Center, Pharmaceutical Technology Institute, Mashhad University of Medical Sciences, Mashhad, Iran; 3 Applied Biomedical Research Center, Mashhad University of Medical Sciences, Mashhad, Iran; 4 Arthropod-Borne Diseases Research Center, Ardabil University of Medical Sciences, Ardabil, Iran

**Keywords:** *Pseudomonas aeruginosa*, Colistin, Multidrug-resistant

## Abstract

**Background:**

*Pseudomonas aeruginosa* is a common cause of nosocomial infections. However, the emergence of multidrug-resistant strains has complicated the treatment of *P. aeruginosa* infections. While polymyxins have been the mainstay for treatment, there is a global increase in resistance to these antibiotics. Therefore, our study aimed to determine the prevalence and molecular details of colistin resistance in *P. aeruginosa* clinical isolates collected between June 2019 and May 2023, as well as the genetic linkage of colistin-resistant *P. aeruginosa* isolates.

**Results:**

The resistance rate to colistin was 9% (*n* = 18) among *P. aeruginosa* isolates. All 18 colistin-resistant isolates were biofilm producers and carried genes associated with biofilm formation. Furthermore, the presence of genes encoding efflux pumps, TCSs, and outer membrane porin was observed in all colistin-resistant *P. aeruginosa* strains, while the *mcr-1* gene was not detected. Amino acid substitutions were identified only in the PmrB protein of multidrug- and colistin-resistant strains. The expression levels of *mexA*, *mexC*, *mexE*, *mexY*, *phoP*, and *pmrA* genes in the 18 colistin-resistant *P. aeruginosa* strains were as follows: 88.8%, 94.4%, 11.1%, 83.3%, 83.3%, and 38.8%, respectively. Additionally, down-regulation of the *oprD* gene was observed in 44.4% of colistin-resistant *P. aeruginosa* strains.

**Conclusion:**

This study reports the emergence of colistin resistance with various mechanisms among *P. aeruginosa* strains in Ardabil hospitals. We recommend avoiding unnecessary use of colistin to prevent potential future increases in colistin resistance.

**Supplementary Information:**

The online version contains supplementary material available at 10.1186/s12866-024-03309-1.

## Background

*Pseudomonas aeruginosa* is a Gram-negative opportunistic pathogen commonly found in hospital environments, particularly in intensive care units (ICUs). It is responsible for various nosocomial infections, including pulmonary, bloodstream, urinary tract, surgical site, and skin and soft tissue infections [1–4]. The treatment of *P. aeruginosa* infections typically involves the use of β-lactam, fluoroquinolone, and aminoglycoside antibiotics either alone or in combination [5, 6]. However, the misuse and overuse of antibiotics have led to the emergence of antibiotic resistance in *P.* aeruginosa strains, making it difficult to effectively treat these infections. The rise in antibiotic resistance among Gram-negative bacteria, which began in the 1970s, continues to be a significant challenge [7–9]. In 2017, the World Health Organization (WHO) declared antimicrobial resistance a global crisis. The organization listed 12 bacterial families as the greatest threats to human health, with carbapenem-resistant *P. aeruginosa* strains being one of the most important [10]. Carbapenem antibiotics are commonly recommended for the treatment of multidrug-resistant (MDR) *P. aeruginosa* strains [8]. However, in line with the WHO report, our previous studies have confirmed the high prevalence of carbapenem-resistant *P. aeruginosa* isolates in various cities of Iran, particularly in Ardabil in the northwest of the country [3, 11]. This challenging situation has increased the reliance on polymyxins, such as polymyxin B and colistin, which are cationic lipopeptide antibiotics and considered as the last resort for treating Gram-negative bacteria, including carbapenem-resistant *P. aeruginosa* strains that are resistant to all other available antibiotics [12]. While colistin (also known as polymyxin E) resistance has not been observed in clinical isolates of *P. aeruginosa* in Ardabil city thus far, colistin-resistant *P. aeruginosa* has been reported in other cities in Iran and worldwide. Therefore, one of the aims of this study was to investigate the resistance of *P. aeruginosa* clinical strains to colistin in Ardabil, as well as their genetic linkage. The mechanisms of polymyxin resistance in Gram-negative bacteria are not fully understood. However, they can be mediated through plasmid-encoded genes, such as the acquisition of the mobilized colistin resistance-1 (*mcr-1*) gene, or through chromosomally encoded genes, including 1) cationic modification of lipid A in lipopolysaccharides (LPS) via two-component systems (TCSs: PhoPQ and PmrAB), 2) loss of LPS, 3) overexpression of efflux pump systems and capsular polysaccharides, 4) down-regulation of porin (OprD), and 5) enzymatic inactivation of colistin [7]. Therefore, another objective of this study was to determine the most common mechanisms of colistin resistance among drug-resistant *P. aeruginosa* clinical isolates collected from patients referred to hospitals in Ardabil, Iran.

## Materials and methods

### *P. aeruginosa* clinical isolates, materials, and equipment

In this cross-sectional study conducted in Iran, a country located southwest of the Asian continent, a total of 200 *P. aeruginosa* clinical isolates were utilized. These strains were collected from various specimens, including urine (*n* = 90), sputum (*n* = 55), wound (*n* = 28), blood (*n* = 26), and cerebrospinal fluid (CSF) (*n* = 1), obtained from patients referred to hospitals in Ardabil city, northwest of Iran. The hospitals included Imam Khomeini (*n* = 105), Alavi (*n* = 55), Imam Reza (*n* = 25), Bu-Ali (*n* = 6), Sabalan (*n* = 6), Fatemi (*n* = 2), and Ghaem (*n* = 1). The data collection period spanned from June 2019 to May 2023.

The initial identification of *P. aeruginosa* clinical isolates was performed using phenotypic standard laboratory tests, which included assessments of pigment production, colony morphology, oxidase, catalase, IMViC pattern, and Gram staining. Confirmation of the species was subsequently achieved using the polymerase chain reaction (PCR) with a specific species primer [3].

The most important materials used in our study were the Master Mix for PCR/ERIC-PCR and real-time PCR (Ampliqon, Denmark), primers (Metabion, Germany), TRIzol™ Reagent (Bio Basic, Ontario, Canada), cDNA synthesis kit (Yekta Tajhiz Azma, Tehran, Iran), Mueller Hinton agar (Conda, Pronasida, Spain), colistin sulfate salt powder (Sigma-Aldrich co, St. Louis, MO, ≥ 15,000 U/mg), antibiotic disks (Padtan Teb, Iran), Cetrimide agar (Conda, Pronasida, Spain), and Trypticase Soy Broth (TSB) (QUELAB/UK). Additionally, the following equipment was used: Eppendorf thermal cycler (Germany), LightCycler® System (Roche Diagnostics), NanoDrop 2000c Spectrophotometer (Thermo Scientific, USA), Agarose Gel Electrophoresis (Padideh Nojen Pars, Iran), and ELISA microplate reader (BioTek, USA).

### Colistin agar test

The susceptibility pattern of *P. aeruginosa* clinical isolates to colistin was determined based on the agar dilution MIC (minimum inhibitory concentration) method on Mueller–Hinton agar as suggested by the Clinical and Laboratory Standards Institute (CLSI) [13]. For this purpose, 3–5 fresh *P. aeruginosa* colonies from Mueller Hinton agar plates were picked and transferred to 4–5 mL sterile saline to prepare 0.5 McFarland turbidity standards. Bacterial standard suspensions were diluted in saline (1:10). A 10 μL of each diluted bacterial suspension was poured onto a colistin agar plate. Colistin agar plates were prepared in required dilutions, *i.e.*, 0.5–16 μg/mL. Incubation condition and length were maintained at 37 °C for 16–18 h. *P. aeruginosa* clinical isolates with MIC values ≥ 4 μg/mL were considered as resistant strains. A colistin-resistant *Acinetobacter baumannii* clinical isolate was used as the positive control (MIC = 16 μg/mL) (Ethics ID: IR.ARUMS.REC.1400.071).

The disk diffusion method was used to determine multiple drug resistance patterns (multidrug-resistant (MDR), extremely drug-resistant (XDR), and pandrug-resistant (PDR) strains) among colistin-resistant *P. aeruginosa* clinical isolates [14]. Furthermore, this method was employed to assess the resistance rates of colistin-resistant *P. aeruginosa* isolates against various antibiotics, including piperacillin (100 μg), piperacillin-tazobactam (100/10 μg), ceftazidime (30 μg), cefepime (30 μg), aztreonam (30 μg), imipenem (10 μg), meropenem (10 μg), gentamicin (10 μg), tobramycin (10 μg), amikacin (30 μg), ciprofloxacin (5 μg), levofloxacin (5 μg), norfloxacin (10 μg), and ofloxacin (5 μg), as per our previous study [3]. *P. aeruginosa* ATCC 27853 was used as a reference strain.

### Biofilm formation assay

Evaluation of biofilm production among colistin-resistant *P. aeruginosa* isolates was performed by a colorimetric assay [15]. For this aim, of the 1:100 diluted suspensions of *P. aeruginosa* isolates which had grown in TSB mediums, 150 μL were inoculated into a sterile 96-well flat bottom plate and incubated at 37 °C for 24 h. The plate was washed with 200 μL of phosphate-buffered saline (PBS) (pH ~ 7.4) three times. Biofilm fixation was done with 100 μL of methanol (99%) for 15 min and then the wells were air-dried. 150 μL of crystal violet stain (1%) was added to wells for 20 min, unbound stain washed with water, and then bound stain released through 150 μL of acetic acid (33%). ELISA microplate reader was used to measure the optical density (OD) of wells at 590 nm. Colistin-resistant *P. aeruginosa* isolates were divided into four categories including no biofilm producer if the OD of a strain (ODs) was less or equal to the OD negative control (ODc), weak biofilm producer if ODc < ODs < 2 × ODc, moderate biofilm producer if 2 × ODc < ODs < 4 × ODc, and strong biofilm producer if 4 × ODc < ODs. *P. aeruginosa* ATCC 27853 and sterile TSB medium were used as positive and negative controls, respectively. All experiments are performed in triplicate.

### Detection of colistin resistance genes

Molecular identification of the genes encoding efflux pumps (i.e., *mexA*, *mexC*, *mexE*, and *mexY* genes), TCSs (i.e., *phoP*, *phoQ*, *pmrA*, and *pmrB* genes), outer membrane porin (*oprD* gene), and *mcr-1* gene, as well as genes involved in biofilm formation of *P. aeruginosa* (i.e., *algD*, *pslD*, *pelF*, *Ppgl*, and *PAPI-1* genes) were performed by the PCR method. Used primers along with the PCR program for the detection of each gene were listed in Table 1. In brief, genomic DNA was extracted from the 200 *P. aeruginosa* clinical isolates by the boiling method [3] and confirmed by a spectrophotometer. Amplification of the genes was performed in a final volume of 15 μL (10 μL of Master Mix, with 3 μL of template DNA (50 ng/µL), and 2 μL of primers (10 μmol/L)) and then their presence was confirmed using the agarose gel electrophoresis and sequencing (Sanger method, Pishgam, Iran) techniques. It is worth mentioning that for some genes with non-specific bands on agarose gel, the values mentioned above along with the PCR conditions were changed. Finally, we employed the enterobacterial repetitive intergenic consensus (ERIC)-PCR method to assess the genetic relatedness among colistin-resistant *P. aeruginosa* isolates. In pursuit of this, amplification reactions were conducted in a final volume of 50 μL using the primers and ERIC-PCR program outlined in Table 1. Subsequently, the ERIC-PCR products were electrophoresed on a 2% agarose gel, and the resulting band patterns were analyzed using the Dice distance method for similarity and the UPGMA analysis method for clustering (GelQuest software version 3.3.5.0). ERIC-PCR band patterns exhibiting > 80% similarity were categorized as belonging to the same cluster [5].

**Table 1:** Used primers along with PCR/ERIC-PCR and qRT-PCR programs

Gene	Oligonucleotide sequence (5′ to 3′)	PCR/ERIC-PCR condition	Amplicon size (bp)	qRT-PCR condition	Reference
*mexA*	F: CCTGCTGGTCGCGATTTCGG R: CCAGCAGCTTGTAGCGCTGG		332		[16]
*mexC*	F: TTGGCTATGGCCATCGCGTT R: ATCGAAGTCCTGCTGGCTGA		390		[16]
*mexE*	F: ATCCCACTTCTCCTGGCGCT R: GGTCGCCTTTCTTCACCAGT		260		[16]
*mexY*	F: CCGCTACAACGGCTATCCCT R: AGCGGGATCGACCAGCTTTC		246		[16]
*oprD*	F: CGACCTGCTGCTCCGCAACTA R: TTGCATCTCGCCCCACTTCAG		301		[17]
*rpsL*	F: GCTGCAAAACTGCCCGCAACG R: ACCGCAGGTGTCCAGCGAACC		250		[16]
*PAPI-1*	F: CATCAACCGGATCGACGAAGT R: GTCAACCCTCTGATCCAAAAAGTT		462		[18]
*pelF*	F: GAGGTCAGCTACATCCGTCG R: TCATGCAATCTCCGTGGCTT		789		[18]
*pslD*	F: TGTACACCGTGCTCAACGAC R: CTTCCGGCCCGATCTTCATC		369		[18]
*Ppgl*	F: GTGGTGGGGACCTATACCGAA R: GTAGTTGGCGACGAACAGGTA		327		[18]
*algD*	F: CGTCTGCCGCGAGATCGGCT R: GACCTCGACGGTCTTGCGGA		313		[3]
*phoP*	F: TTGCGCCACCACCTCTATAC R: GAACTGGAACGGCTTGACC		282		(This study)
*phoQ*	F: GCAACGAATTCCACACCAC R: GAATCGTCCAGGCTCAGTTC		964		(This study)
*pmrA*	F: GACCAAGCCCTTCGATCTC R: AGGTGGTGGACGTGGACTT		294		(This study)
*pmrB*	F: CCTACCACCTCTCGCTGAAG R: GAAGTGCAGTTCGACGATGC		1211		[19]
*mcr-1*	F: CGGTCAGTCCGTTTGTTC R: CTTGGTCGGTCTGTAGGG		309		[19]
*ERIC*	F: ATGTAAGCTCCTGGGGATTCAC R: AAGTAAGTGACTGGGGTGAGCG		100–1700		[20]

### Mutational analysis of the PhoPQ and PmrAB

Detection of colistin resistance-associated mutations among *P. aeruginosa* clinical isolates with multiple drug resistance was performed using the sequencing method. The PCR products of the *phoQ* and *pmrB* genes from *P. aeruginosa* clinical isolates MDR, XDR, and resistant to colistin were sent for sequencing. The nucleotide sequences were compared with colistin-susceptible *P. aeruginosa* reference strain ATCC 27853 using the BioEdit software (version 7.2.5). Additionally, an online data analysis service (available at https://web.expasy.org/translate/) was utilized to assess amino acid alterations.

### Expression of the genes encoding efflux pumps, TCSs, and outer membrane porin

Expression levels of the *mexA*, *mexC*, *mexE*, *mexY*, *phoP*, *pmrA*, and *oprD* genes were determined among resistant isolates using the quantitative reverse transcription PCR (qRT-PCR) and specific sets of primers (Table 1). In brief, the total RNA of colistin-resistant *P. aeruginosa* isolates was extracted using the TRIzol™ Reagent. After confirming the quality and quantity of extracted RNAs (1 μg), cDNA synthesis was done according to the manufacturer’s instructions. The qRT-PCR of the genes was carried out under conditions presented in Table 1 and in a final volume of 15 μL (SYBR Green PCR Master Mix (7 μL), primers (2 μL, 10 μmol/L), cDNA (1 μg/μL), and DEPC-treated water (5 μL)). The 30S ribosomal *rpsL* gene was used as the normalizing gene. The expression levels of genes in colistin-resistant *P. aeruginosa* isolates were determined relative to their expression levels in *P. aeruginosa* ATCC 27853 using the 2^−ΔΔCt^ method. Expression for each gene was assessed in duplicate.

Interpretation of the results of qRT-PCR was performed as follows: for the *mexA* and *mexC* genes; twofold, for the *mexE* gene; tenfold, and for the *mexY* gene; fourfold expression rates compared with the reference strain of *P. aeruginosa* ATCC 27853 were considered as overexpression [16]. For the *oprD* gene, the expression rate ≤ 30% relative to the reference strain was considered as down-regulation [17]. In addition, for the *phoP* and *pmrA* genes, expression levels higher than those of *P. aeruginosa* ATCC 27853 were considered as increased gene expression [12].

## Result

**Fig. 1:**
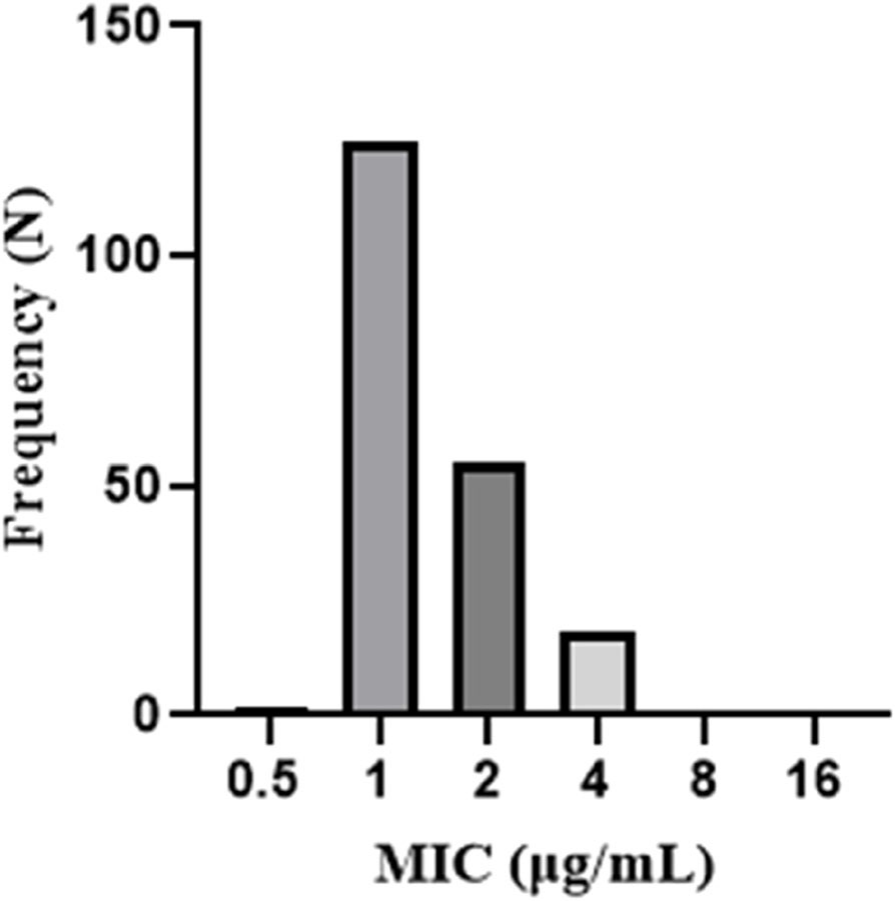
MIC values of colistin for 200 clinical isolates of *P. aeruginosa*

Among 200 *P. aeruginosa* clinical isolates obtained from hospitalized patients, 18 isolates (9%) were resistant to colistin antibiotic according to the agar dilution method. Characteristics of these 18 colistin-resistant *P. aeruginosa* isolates were presented in Table 2. In addition, MIC values of colistin for clinical isolates of *P. aeruginosa* are reported in Fig. 1. Among 200 *P. aeruginosa* strains, MIC values of 0.5, 1, 2, and 4 μg/mL were found in 2 (1%), 125 (62.5%), 55 (27.5%), and 18 (9%) isolates, respectively. All colistin-resistant isolates showed MIC = 4 μg/mL.

**Table 2:** Mechanisms of colistin resistance among 18 colistin-resistant *P. aeruginosa* clinical isolates

Isolate number	Type of specimen	Hospital	Resistance type	Biofilm production	Resistance mechanisms								
					Amino acid alteration		Two-component systems overproduction		Porin down-regulation	Efflux pumps overproduction			
					*phoQ*	*pmrB*	*phoP*	*pmrA*	*oprD*	*mexA*	*mexC*	*mexE*	*mexY*
8	Blood	Imam Khomeini		Moderate			**4.4**	**45.5**	2.2	**14.5**	**88.6**	6.7	**15.7**
24	Urine	Alavi	XDR	Weak	No change	Tyr345His	**1.8**	0.0	**0.0**	**4.1**	**41.3**	0.0	**6.8**
45	Urine	Bu-Ali		Weak			**5.7**	0.0	**0.2**	**6.4**	**55.7**	0.0	**9.3**
46	Blood	Bu-Ali		Weak			**4.1**	0.0	0.4	**6.9**	**71.5**	0.0	**12.7**
51	Urine	Alavi		Weak			**3.5**	0.0	**0.3**	**2.8**	**15.7**	0.0	**6.1**
71	Urine	Alavi		Strong			**14.6**	**64.8**	5.5	**14.7**	**136.2**	**10**	**19**
72	Urine	Imam Reza	MDR	Weak	No change	Tyr345His	1	0.0	**0.0**	0.7	**5.2**	0.0	1
76	Urine	Imam Reza		Weak			**1.5**	0.0	0.6	**5**	**67.1**	0.0	**9.9**
79	Urine	Imam Reza	MDR	Moderate	No change	Tyr345His	**6.6**	0.0	**0.0**	**9**	**55.7**	0.0	**23.9**
80	Wound	Imam Khomeini	MDR	Strong	No change	Tyr345His	**9.6**	0.0	0.5	**68.1**	**119.4**	0.0	**10.3**
81	Urine	Imam Khomeini	MDR	Moderate	No change	Tyr345His	**4.7**	0.0	0.9	**63.1**	**51.9**	0.0	**3.9**
82	Urine	Imam Reza		Moderate			**14.5**	**18.7**	60.5	**35.7**	**34.2**	8	**30.9**
89	Urine	Imam Khomeini		Strong			**8.8**	**45.8**	0.4	**36.2**	**89.8**	4	**21.4**
91	Urine	Imam Khomeini		Moderate			**3.5**	**11.5**	**0.0**	**10.7**	**29.8**	0.0	**6.7**
92	Urine	Imam Khomeini		Moderate			**4.3**	0.0	6.1	**5.3**	**28.4**	0.0	**11.1**
117	Urine	Sabalan		Moderate			1	**33.3**	**0.0**	1.2	0.4	0.0	1.6
141	Sputum	Imam Reza	XDR	Moderate	No change	Tyr345His	1	**6.4**	**0.1**	**2.2**	**24.2**	3	3.4
143	Wound	Imam Khomeini		Weak			**12**	0.0	0.7	**22.1**	**151.1**	**11.7**	**40.7**
*P. aeruginosa* ATCC 27853							1	1	1	1	1	1	1

^a^Overexpressed values *phoP*, *pmrA*, *mexA*, *mexC*, *mexE*, *mexY*, and down-regulated values of *oprD* were indicated in bold

Of the 18 colistin-resistant *P. aeruginosa* strains, 6 strains showed multiple drug resistance patterns (4 MDR and 2 XDR) in the disk diffusion method. The susceptibility patterns of colistin-resistant *P. aeruginosa* strains to various antibiotics along with their virulence gene profiles are presented in Table 3. The resistance rates of 18 colistin-resistant *P. aeruginosa* isolates to different antibiotics were as follows: piperacillin 11.1%, piperacillin-tazobactam 5.5%, ceftazidime 11.1%, cefepime 11.1%, aztreonam 0%, imipenem 38.9%, meropenem 11.1%, gentamicin 11.1%, tobramycin 11.1%, amikacin 5.5%, ciprofloxacin 50%, levofloxacin 50%, norfloxacin 50%, and ofloxacin 55.5%.

**Table 3:** Profiles of antibiotic resistance and virulence genes of 18 colistin-resistant *P. aeruginosa* clinical isolates

Isolate number	Antibiotic resistance profiles^a^																		Virulence gene profiles^a^
	Phenotypic																	Genotypic	
	Colistin agar test MIC (μg/mL)	Disk diffusion															Combined test		
		PIP	TZP	CAZ	FEP	ATM	IMP	MEM	GEN	TOB	AMK	CIP	LVX	NOR	OFX	MDR/XDR/ TDR			
8	4	S	S	S	S	S	S	S	S	S	S	S	S	S	S		AmpC	*qacEΔ1*, *fabV*, *cepA*	*algD*, *plcN*, *lasB*, *plcH*, *exoS*, *toxA*, *pilB*
24	4	R	I	R	R	I	R	R	R	R	R	R	R	R	R	XDR	MBL	*gyrA*, *gyrB*, *parE*, *parC*, *qacEΔ1*, *IMP*, *TEM*, *oxa-2*, *oxa-10*, *intI-1*	*algD*, *plcN*, *lasB*, *plcH*, *toxA*, *exoU*
45	4	S	S	S	S	S	S	S	S	S	S	S	S	S	S		AmpC	*qacEΔ1*, *fabV*, *cepA*, *oxa-2*, *TEM*	*algD*, *plcN*, *lasB*, *plcH*, *toxA*, *exoU*
46	4	S	S	S	S	S	S	S	S	S	I	S	S	S	S		AmpC	*fabV*, *cepA*, *oxa-2*, *TEM*	*algD*, *plcN*, *lasB*, *plcH*, *exoS*, *toxA*, *exoU*
51	4	1	S	S	S	S	I	S	S	S	I	S	S	S	S		AmpC	*qacEΔ1*, *fabV*, *cepA*	*algD*, *plcN*, *lasB*, *plcH*, *exoS*, *toxA*, *exoU*
71	4	S	S	S	S	S	R	S	S	S	S	S	S	S	S		MBL	*oxa-23*	*algD*, *plcN*, *lasB*, *plcH*, *exoS*, *toxA*, *exoU*, *pilB*
72	4	S	S	S	S	S	R	S	S	S	S	R	R	R	R	MDR	MBL	*qacEΔ1*, *qacE*, *fabV*, *cepA*, *oxa-2*, *PSE*, *intI-1*	*algD*, *plcN*, *lasB*, *plcH*, *toxA*, *exoU*, *pilB*
76	4	I	S	S	S	S	I	S	S	S	S	R	R	R	R		AmpC	*oxa-23*, *PSE*, *intI-1*	*algD*, *plcN*, *lasB*, *plcH*, *toxA*, *exoU*, *pilB*
79	4	I	I	I	S	S	R	S	S	S	S	R	R	R	R	MDR	MBL AmpC ESBL	*qacEΔ1*, *qacE*, *qacG*, *fabV*, *cepA*, *oxa-2*, *oxa-23*, *intI-1*	*algD*, *plcN*, *lasB*, *plcH*, *toxA*, *exoU*, *pilB*
80	4	S	S	S	S	S	R	S	S	S	S	R	R	R	R	MDR	MBL AmpC	*fabV*, *cepA*, *oxa-2*, *oxa-23*, *PSE*, *intI-1*	*algD*, *plcN*, *lasB*, *plcH*, *toxA*, *exoU*, *pilB*
81	4	S	S	S	S	I	R	S	S	S	I	R	R	R	R	MDR	MBL AmpC	*oxa-2*, *oxa-23*, *PSE*, *intI-1*	*algD*, *plcN*, *lasB*, *plcH*, *toxA*, *pilB*
82	4	I	S	S	S	I	S	S	S	S	S	S	S	S	S			*oxa-23*, *PSE*	*algD*, *plcN*, *lasB*, *plcH*, *exoS*, *toxA*, *pilB*
89	4	S	S	S	S	S	S	S	S	S	S	S	S	S	S			*oxa-2*, *oxa-23*	*algD*
91	4	S	S	S	S	S	S	S	S	S	S	R	R	R	R			*oxa-2*, *oxa-10*, *oxa-23*, *PSE*,* intI-1*	*algD*
92	4	S	S	S	S	S	I	S	S	S	S	R	R	R	R		AmpC	*oxa-2*, *oxa-10*, *oxa-23*, *PSE*,* intI-1*	*algD*
117	4	S	S	S	S	S	I	S	S	S	S	S	S	S	S		AmpC	*oxa-2*, *PSE*	*algD*
141	4	I	I	R	R	I	R	R	R	R	S	R	R	R	R	XDR	ND	ND	*algD*
143	4	R	R	S	I	S	I	I	S	S	S	S	S	S	I		ND	ND	*algD*

*Abbreviations: PIP* Piperacillin, *TZP* Piperacillin-Tazobactam, *CAZ* Ceftazidime, *FEP* Cefepime, ATM Aztreonam, *IMP* Imipenem, *MEM* Meropenem, *GEN *Gentamicin, *TOB* Tobramycin, *AMK* Amikacin, *CIP* Ciprofloxacin, LVX: Levofloxacin, NOR: Norfloxacin, *OFX* Ofloxacin, R: Resistant, *S* Susceptible, *I* Intermediate, *ESBL* Extended-spectrum β-lactamase, *MBL* Metallo-β-lactamase, *AmpC* AmpC cephalosporinase, *ND* Not determined

^a^Presented data on profiles of antibiotic resistance and virulence genes are based on previous studies

**Fig. 2:**
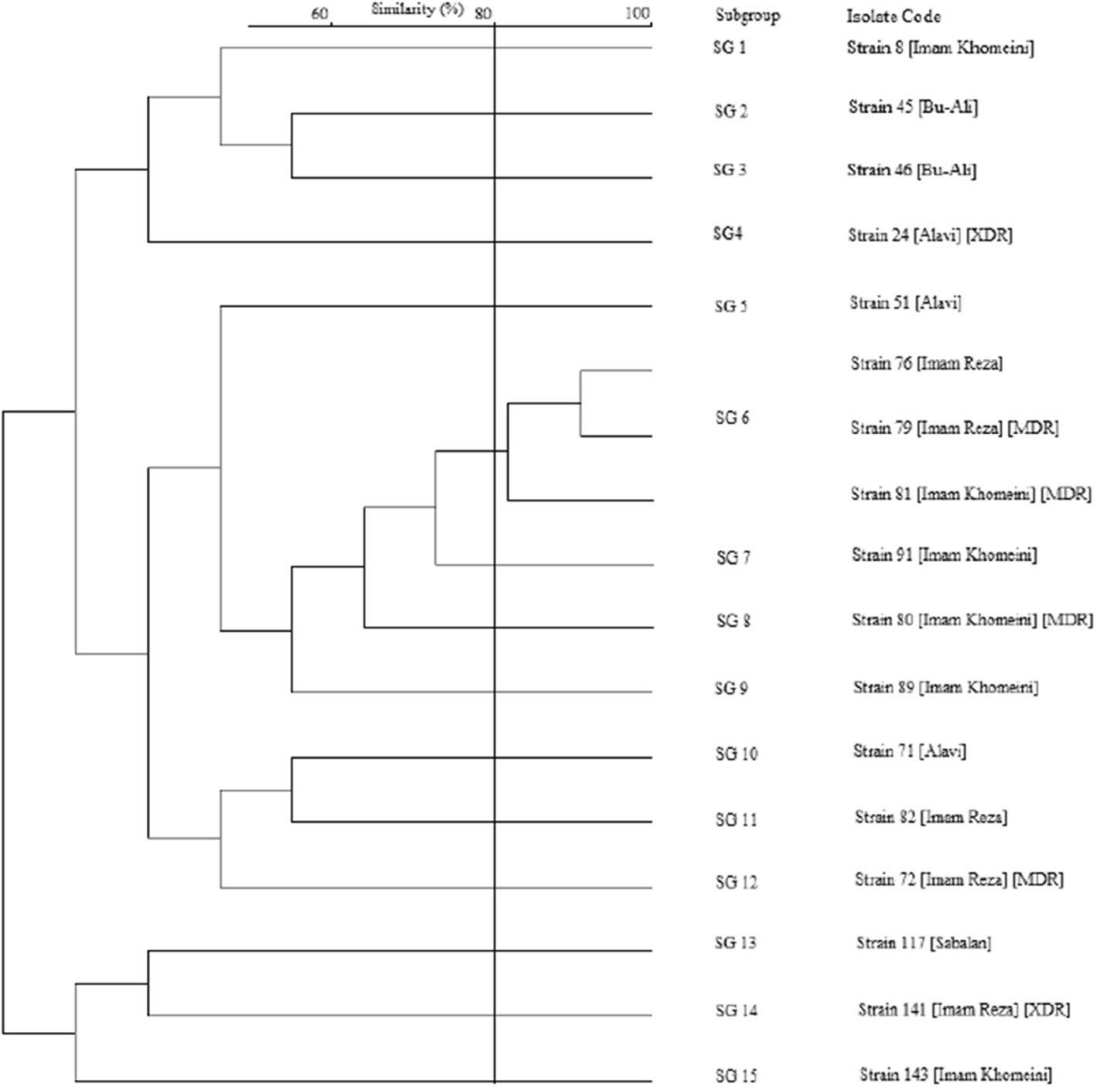
Dendrogram of colistin-resistant *P. aeruginosa* clinical isolates based on the ERIC-PCR band patterns

A high genetic diversity was observed among the 18 colistin-resistant *P. aeruginosa* clinical isolates by detecting 15 different ERIC-PCR band patterns (Fig. 2). Among tested strains, 15 colistin-resistant *P. aeruginosa* exhibited unique genotypes (subgroup), while genotype subgroup 6 comprised three isolates. Details of band patterns for each species are depicted in Supplementary Figure S1.

As shown in Table 3, colistin-resistant *P. aeruginosa* strains were carrying the genes encoding resistance to other antibiotics such as metallo-β-lactamase (*IMP* gene), AmpC cephalosporinase, extended-spectrum β-lactamase (*TEM* and *PSE* genes), oxacillinase (*oxa-2* and *oxa-23* genes), and efflux pumps (*qacEΔ1*, *qacE*, *qacG*, and *cepA* genes). These genes are involved in the emergence of MDR and XDR *P. aeruginosa* strains.

Biofilm formation was identified in all 18 colistin-resistant *P. aeruginosa* isolates in the colorimetric assay. Among them, 7 (38.9%) isolates were weak biofilm producers, 8 (44.4%) isolates were moderate biofilm producers, and 3 (16.7%) isolates were strong biofilm producers. In addition, the presence of genes encoding biofilm (*i.e., algD*, *pslD*, *pelF*, *Ppgl*, and *PAPI-1* genes) was detected in all 18 colistin-resistant *P. aeruginosa* isolates in the PCR and confirmed by sequencing. The GenBank accession numbers for our nucleotide sequences of detected genes in this study are OR855380 to OR855387.

The role of the *phoQ* and *pmrB* gene mutations in the emergence of colistin-resistant *P. aeruginosa* was evaluated in 6 MDR and XDR clinical isolates. Analysis of the *phoQ* gene revealed the following nucleotide substitutions: at positions 473 C → T, 495 G → A, 552 T → C, 583 T → C, 675 T → G, 702 A → G, 1110 C → T, 1146 C → T, and 1155 C → T. None of these nucleotide substitutions led to amino acid alterations. Furthermore, similar results were observed for the *pmrB* gene and nucleotide changes did not result in amino acid substitutions except for Tyr345His. Nucleotide substitutions of the *pmrB* gene were as follows: G at position 507 to A, G at position 639 to A, A at position 645 to G, G at position 690 to C, T at position 696 to C, A at position 750 to G, A at position 762 to G, C at position 891 to T, and T at position 1033 to C.

Expression levels of the *mexA*, *mexC*, *mexE*, *mexY*, *phoP*, and *pmrA* genes in 18 colistin-resistant *P. aeruginosa* strains were as follows: 88.8% (*n* = 16), 94.4% (*n* = 17), 11.1% (*n* = 2), 83.3% (*n* = 15), 83.3% (*n* = 15), and 38.8% (*n* = 7), respectively. In addition, down-regulation of the *oprD* gene was observed in 44.4% (*n* = 8) of colistin-resistant *P. aeruginosa* strains. The presence of a plasmid-borne *mcr-1* gene and its association with colistin resistance in *P. aeruginosa* strains was not confirmed in this study.

## Discussion

In recent years, there has been a significant reduction in the susceptibility of *P. aeruginosa* to polymyxins, despite its inherent sensitivity to these antibiotics [21]. The current study reported, for the first time, a prevalence of 9% for colistin-resistant *P. aeruginosa* in Ardabil city. Other studies conducted in different regions of Iran and abroad have reported varying rates of colistin resistance among *P. aeruginosa* isolates. These include studies by Abd El-Baky et al. in Egypt (21.3%) [22], Rossi et al. in Brazil (6.3%) [23], Wi et al. in South Korea (7.4%) [24], Zarate et al. in Peru (7.2%) [25], Farajzadeh Sheikh et al. in Iran (Ahvaz, Tehran, and Isfahan) (1.3%) [26], Goli et al. in Iran (Tabriz) (2%) [27], Heidari et al. in Iran (Isfahan and Shiraz) (7%) [28], Malekzadegan et al. in Iran (Shiraz) (0%) [29], Tahmasebi et al. in Iran (Hamadan) (3.9%) [30], and Talebi et al. in Iran (Tehran) (0%) [19].

The variations in colistin resistance rates among *P. aeruginosa* isolates in different regions can be attributed to several factors. These include differences in sample size, methods used for antimicrobial susceptibility testing, and specific local factors. In the case of Ardabil, the higher rate of colistin resistance compared to other cities in Iran may be attributed to three main factors. Firstly, the use of colistin in veterinary medicine for the growth promotion of food-producing animals, as Ardabil is an agricultural and animal husbandry province [31]. This practice can contribute to the selection and spread of colistin-resistant strains. Secondly, the misuse of antibiotics in intensive care units, which can lead to the emergence of resistant strains [22]. It's worth noting that Iran has been reported to have the second-highest antibiotic consumption rate in the world, with antibiotic consumption 16 times higher than the global standard (mehrnews.com/xYQDx), as stated by the secretary of the Infectious Diseases Association of Iran. Thirdly, while the transmission of colistin-resistant *P. aeruginosa* within hospitals or across the entire healthcare system has been a concern [32], our investigation revealed no genetic correlation among colistin-resistant strains collected from different hospitals in Ardabil city. However, we did observe three strains in genotype subgroup 6 that were shared between two distinct hospitals (Fig. 2). A similar finding was reported by Khosravi et al. for MDR *P. aeruginosa* isolates in Ahvaz [33]. In contrast, Zarei et al. identified clonal relatedness between clinical and environmental *P. aeruginosa* isolates [20].

Regarding the emergence of colistin-resistant *P. aeruginosa* strains in Ardabil hospitals, various bacterial factors may be involved. One such factor is biofilm formation. A biofilm is a bacterial population encased in an outer polymer layer, consisting of host immune system products or bacterial secreted polymers like exopolysaccharides (EPS), extracellular DNA, and proteins. Biofilms play a role in acute burn wound infections [1, 34]. Studies have shown that extracellular DNA within the biofilm, along with factors such as magnesium or calcium starvation, low pH, and antimicrobial peptides (including colistin), can contribute to polymyxin resistance through modifications in LPS via the activation of TCSs like PmrAB and PhoPQ [34, 35]. Our study's findings are consistent with the previous research discussed above. We observed that all colistin-resistant strains of *P. aeruginosa* in our study were capable of producing biofilms, as indicated in Table 2. This ability poses a significant challenge in the treatment of *P. aeruginosa* infections, particularly those affecting wounds. Furthermore, the presence and excessive production of alginate EPS within the biofilm provide *P. aeruginosa* with protection against phagocytic cells and antibiotic treatments [34]. Apart from the *algD* gene, which encodes alginate, we identified the presence of other genes associated with biofilm formation (*pslD*, *pelF*, *Ppgl*, and *PAPI-1*) in all colistin-resistant *P. aeruginosa* isolates. However, the prevalence of these chromosomal genes differed from a study conducted by Rajabi in Iran, where the respective prevalence rates were as follows: *algD* 78.6%, *pelF* 70.5%, *pslD* 36.6%, *Ppgl* 0%, and *PAPI-1* 77.6% [18]. Notably, the presence of the *PAPI-1* gene in all *P. aeruginosa* isolates in our study supports the findings of Qiu et al., which suggest that this large pathogenicity island can be transmitted between *P. aeruginosa* strains [36].

Modifications of the negatively charged phosphate groups of lipid A through adding phosphoethanolamine mediated by the *mcr* gene can lead to polymyxins resistance [35]. However, our study did not find evidence of the involvement of this plasmid-borne gene in the emergence of colistin-resistant *P. aeruginosa* strains. Similar results were obtained in Tabriz, Iran, where all colistin-resistant Gram-negative isolates, including *P. aeruginosa* strains, tested negative for *mcr* genes [37]. Additionally, another study conducted in Ardabil on clinical isolates of colistin-resistant *A. baumannii* also demonstrated the absence of this gene (data not published). One possible explanation for the 0% prevalence of the *mcr-1* gene in our study is its association with *Enterobacteriaceae*, particularly *Escherichia coli*, which are resistant to colistin and commonly isolated from animal sources [37].

Multiple studies have confirmed that mutations in the components of TCSs, namely PmrB and PhoQ proteins, play a significant role in the development of polymyxin resistance in *P. aeruginosa* strains. These mutations result in the upregulation of the *arnBCADTEF* operon, leading to the substitution of phosphate groups of lipid A with the cationic 4-amino-4-deoxy-L-arabinose in the LPS structure [38]. Several different mutations have been reported in various studies; however, in our study, we observed only the amino acid alteration Tyr345His in the sensor kinase protein PmrB, a component of the PmrAB TCS. This amino acid alteration has also been reported in other studies conducted by Barrow et al. [39], Sellera et al. [40], Lee et al. [41], and Schurek et al. [42]. It appears that the Tyr345His substitution in the PmrB protein is not involved in the activation of the response regulator PmrA through phosphorylation in colistin-resistant *P. aeruginosa* strains isolated from hospitals in Ardabil. As shown in Table 2, except for strain 141, colistin-resistant *P. aeruginosa* strains containing the Tyr345His mutation did not exhibit overproduction of the *pmrA* gene. In our study, nucleotide substitutions in the *phoQ* gene did not result in amino acid alterations. Therefore, similar to the *pmrB* gene, there is no association between mutations in the *phoQ* gene and subsequent overproduction of the *phoP* gene in the emergence of colistin-resistant *P. aeruginosa* strains. The overproduction of *PmrB* and *PhoP* genes among colistin-resistant *P. aeruginosa* strains may be attributed to factors other than mutations, such as low levels of magnesium or calcium, low pH, and antimicrobial peptides [34, 35].

Table 2 provides evidence that the development of colistin resistance in *P. aeruginosa* strains in Ardabil hospitals is a result of multiple factors. Previous reports have suggested that the ParRS TCS in *P. aeruginosa* is also involved in the emergence of polymyxin-resistant strains by down-regulating the expression of the porin protein OprD [43]. In this study, this resistance mechanism was confirmed in 44.4% of colistin-resistant *P. aeruginosa* strains. Furthermore, mutations in the ParRS TCS lead to low to moderate levels of resistance to polymyxins by enhancing the production of the MexXY/OprM efflux pump [44]. It is noteworthy that a significant production of efflux pumps, compared to other resistance mechanisms, was observed in 18 colistin-resistant *P. aeruginosa* strains in the current study: MexAB-OprM 88.8%, MexCD-OprJ 94.4%, MexEF-OprN 11.1%, and MexXY-OprM 83.3%. Goli et al. also demonstrated increased expression of genes encoding the MexAB-OprM and MexXY-OprM efflux pumps in two colistin-resistant *P. aeruginosa* strains [27].

### Limitation of the study

In the current research, there were the following limitations due to insufficient resources: 1) the mutations in the *pmrB* and *phoQ* genes were not assessed in all colistin-resistant *P. aeruginosa* isolates, 2) the role of other TCSs (such as ParRS) in the emergence of polymyxin-resistant strains was not studied. And, 3) all variants of *mcr* (including *mcr-2* to *-9*) gene were not investigated.

## Conclusion

The detection of colistin resistance among clinical isolates of *P. aeruginosa* in Ardabil hospitals, higher than in other cities in Iran, is a significant finding. Our study suggests that this resistance can be attributed to various mechanisms, including amino acid alterations in TCSs, overproduction of TCSs, down-regulation of porin, and overproduction of efflux pumps. These results indicate that there may be insufficient infection control measures in Ardabil hospitals and a potential issue with the indiscriminate use of colistin in both humans and animals, which can complicate the treatment of *P. aeruginosa* infections. Therefore, it is recommended to avoid the unnecessary use of this antibiotic to prevent the potential increase in colistin resistance in the future.

### Supplementary Information

**Supplementary Material 1.** 

## Data Availability

The datasets generated and analyzed during the current study are available in the NCBI GenBank repository, under the accession numbers: OR855380 to OR855387

## Abbreviations

*P. aeruginosa*: *Pseudomonas aeruginosa*
ATCC: American Type Culture Collection
CLSI: Clinical and Laboratory Standards Institute
WHO: World Health Organization
MDR: Multidrug-Resistant
XDR: Extremely Drug-Resistant
PDR: Pandrug-Resistant
ICUs: Intensive Care Units
MIC: Minimum Inhibitory Concentration
DNA: Deoxyribonucleic Acid
PCR: Polymerase Chain Reaction
RNA: Ribonucleic Acid
qRT-PCR: Real-Time Quantitative Reverse Transcription PCR
LPS: Lipopolysaccharide
TCSs: Two-Component Systems
PBS: Phosphate-Buffered Saline
OD: Optical Density
ESBL: Extended-spectrum β-lactamase
MBL: Metallo-β-lactamase
AmpC: AmpC cephalosporinase
PIP: Piperacillin
TZP: Piperacillin-Tazobactam
CAZ: Ceftazidime
FEP: Cefepime
ATM: Aztreonam
IMP: Imipenem
MEM: Meropenem
GEN: Gentamicin
TOB: Tobramycin
AMK: Amikacin
CIP: Ciprofloxacin
LVX: Levofloxacin
NOR: Norfloxacin
OFX: Ofloxacin
R: Resistant
S: Susceptible
I: Intermediate
ND: Not determined
