# Supplementary material for: Prevalence and molecular characterization of colistin resistance in Pseudomonas aeruginosa isolates: insights from a study in Ardabil hospitals
Source: BMC Microbiol. 2024 May 3;24:152. doi: 10.1186/s12866-024-03309-1 (PMC11067120; doi:10.1186/s12866-024-03309-1)
Supplement: Supplementary file 1 — Supplementary Material 1. [file 12866_2024_3309_MOESM1_ESM.docx]

**
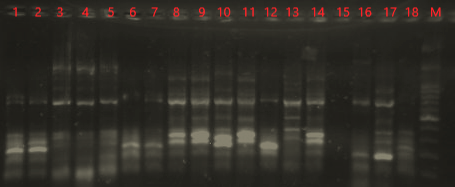
**

**Figure S1.** An image of ERIC-PCR band patterns. Lanes 1-18: Colistin-resistant *P. aeruginosa* isolates, Lane M: Ladder (100 bp).
